# Independent predictors of secondary invasive pancreatic remnant tumors after initial resection of an intraductal papillary mucinous neoplasm: a nationwide large-scale survey in Japan

**DOI:** 10.1007/s00595-020-02074-8

**Published:** 2020-07-13

**Authors:** Yutaka Takigawa, Minoru Kitago, Junichi Matsui

**Affiliations:** 1 Department of Surgery, Tokyo Dental College Ichikawa General Hospital, 5-11-13, Sugano, Ichikawa, Chiba 272-8513 Japan; 2 Department of Surgery, School of Medicine, Keio University, Shinjuku, Tokyo Japan

**Keywords:** Intraductal papillary mucinous neoplasm, Redo pancreatectomy, Secondary pancreatic remnant tumor

## Abstract

**Purpose:**

There is no standardized surveillance protocol after intraductal papillary mucinous neoplasm (IPMN) resection. We report the findings of a large-scale survey in Japan, investigating the independent predictors of secondary invasive tumors by analyzing the epidemiology of secondary tumors of the remnant pancreas after initial IPMN resection.

**Methods:**

An institutional questionnaire about the remnant pancreas after pancreas resection was distributed at the 41st Annual Meeting of the Japanese Society of Pancreatic Surgery in Tokyo. We retrospectively analyzed the patient data including pathological diagnosis, postoperative outcomes, and evaluation methods.

**Results:**

Redo pancreatectomy was performed for secondary disease in 213 (1.4%) of a total 15,777 patients. Eighty-eight of these 213 patients had undergone initial resection of IPMN. The types of secondary tumors after IPMN resection significantly depended on those of the primary tumors. Through short-interval and long-term follow-up, most of the secondary tumors were detected within 1–4 years. Logistic regression analysis revealed that the initial pathological diagnosis of invasive IPMN was an independent predictor of secondary invasive tumors in the remnant pancreas.

**Conclusion:**

Primary invasive IPMN proved to be a significant predictor of secondary invasive IPMN. Both short-interval and long-term follow-up may help to determine the prognosis of patients after IPMN resection.

## Introduction

The ability to detect intraductal papillary mucinous neoplasms (IPMNs) has improved with advances in high-quality diagnostic imaging, which enable the detailed evaluation of patients, post-resection of IPMN [1]. Surgical outcomes, including long-term survival after pancreatectomy, have also improved through surgical and pharmacological advances. Nonetheless, patients who have undergone pancreatic resection for IPMN may be at risk of a spectrum of conditions, ranging from benign non-invasive IPMN to invasive IPMN with a malignant invasive component, synchronous and metachronous multifocal tumor development in the pancreatic parenchyma, and the simultaneous development of pancreatic ductal adenocarcinoma (PDAC) [2–5]. In 2013, European experts issued consensus statements about postoperative IPMN surveillance [6], and in 2012 and 2015, the International Association of Pancreatology (IAP) and the American Gastroenterological Association (AGA), respectively, published guidelines for the management of IPMN patients [7, 8]. The international guidelines from the IAP were subsequently revised in 2017 [9]. Nonetheless, the level of evidence within these reports is fragmentary due to the lack of large-scale cohort studies on the occurrence and recurrence of secondary tumors after IPMN resection. Although retrospective studies have been conducted on secondary tumors after IPMN resection, which support the above guidelines and statements, they are limited by the low number of cases of recurrence [10–12]. Thus, further investigations are necessary to establish a standardized postoperative follow-up protocol for patients who have undergone IPMN resection. This can be improved by detailed data accumulation and analysis of the limited diseases in this field.

Several studies have demonstrated favorable outcomes after repeated resection of pancreatic remnants for secondary tumors including recurrent IPMN [10, 13, 14] and PDAC [15–17]. Unfortunately, in Japan, there is a limited database for the analysis of secondary tumors after IPMN resection, and no established postoperative protocol. We report the findings of our nationwide investigation based on an institutional questionnaire distributed at the 41st Annual Meeting of the Japanese Society of Pancreatic Surgery (JSPS), regarding the independent predictors of secondary invasive tumors through an analysis of the epidemiological and pathological features of secondary tumors of the remnant pancreas after initial resection of IPMN.

## Materials and methods

### Study design

We distributed an institutional questionnaire about the remnant pancreas after IPMN resection at the 41st Annual Meeting of the JSPS in Tokyo, in 2014. We defined “redo pancreatectomy” as repeat resection for a secondary tumor in the remnant pancreas after initial pancreatectomy. The questionnaire consisted of several questions about the number of pancreatectomies, the surveillance system for the pancreatic remnant after the initial pancreatectomy, and cases of redo pancreatectomy between January, 2009 and December, 2013. Responses were collected from 91 institutions affiliated with the JSPS. The question of surveillance after the initial pancreatectomy included an interval of evaluation, a follow-up period, and imaging modalities such as computed tomography and magnetic resonance imaging. Patients were excluded if written informed consent was not obtained or if they had undergone the initial pancreatectomy for tumors other than IPMN. This study protocol was approved by the ethics committee of Tokyo Dental Collage Ichikawa General Hospital (#I14-12, May 02, 2014).

### Study variables

Patient characteristics, the initial surgical procedure, pathological diagnosis at the initial surgery, and postoperative outcomes including complications such as pancreatitis, diabetes mellitus (new onset or worse) or dilatation of the main pancreatic duct (MPD)  (> 2 mm larger in diameter than the preoperative measurement), secondary surgical procedure, and pathological diagnosis at redo pancreatectomy, were collected. We focused not only on the number and timing of secondary tumors such as non-invasive/invasive IPMN and PDAC, but also on the relationship between the primary and secondary tumors.

### Statistical analysis

To identify the independent predictors of invasive IPMN or PDAC, the predictive dependent variables found to be significant in univariate and multivariate analyses were analyzed by logistic regression analysis. After univariate logistic regression analysis of 11 predictive dependent variables, those variables found to be significant or to show a trend toward being an independent predictor were analyzed by multivariate logistic regression analysis. Differences in timing of the development of each tumor (non-invasive IPMN, invasive IPMN, and PDAC) were analyzed by the Kruskal–Wallis test. A *p* value < 0.05 was considered significant. All statistical analyses were carried out by JMP 15.1 for Windows (SAS Institute, Cary, NC, USA).

## Results

### Initial postoperative characteristics

A total of 15,777 pancreatectomies performed at 91 institutions in Japan were recorded between 2009 and 2013. Redo pancreatectomy was performed for secondary disease in 213 (1.4%) of these patients. Written informed consent was obtained from all but one patient. We analyzed, retrospectively, 88 redo pancreatectomy patients who underwent the initial resection for IPMN. Table 1 summarizes the baseline characteristics of these 88 patients (56 men and 32 women; average age, 67.5 ± 7.3 years; range, 54–88 years). The initial pathological diagnosis was non-invasive IPMN in 51 patients and invasive IPMN in 37 patients. Complications after the initial operation included main pancreatic duct dilatation (*n* = 53, 60.2%), postoperative pancreatitis (*n* = 8, 9.1%), and new-onset or worsening diabetes (*n* = 55, 62.5%).

**Table 1:** Patients’ characteristics at the initial pancreatectomy

	*n* = 88
Age, years, mean (± SD)	67.5 (± 7.3)
Male gender, *n* (%)	56 (63.6)
Location of the tumor, *n* (%)	
Pancreatic head	31 (35.2)
Pancreatic body/tail	57 (64.8)
Initial pancreatectomy, *n* (%)	
Proximal	31 (35.2)
Distal	52 (59.1)
Central	5 (5.7)
Pathology of the primary tumor, *n* (%)	
Non-invasive IPMN	51 (58.0)
Invasive IPMN	37 (42.0)
Residual disease in the remnant pancreas, *n* (%)	
IPMN	30 (34.1)
Positive margin	5 (5.7)
None	50 (56.8)
Unknown	3 (3.4)

*IPMN* intraductal papillary mucinous neoplasm, *SD* standard deviation

### Pathological assessment between the primary and secondary tumors

**Fig. 1:**
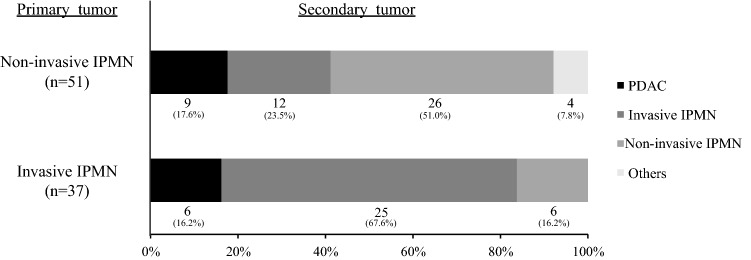
Pathological findings of the primary and secondary tumors. *IPMN* intraductal papillary mucinous neoplasm, *PDAC* pancreatic ductal adenocarcinoma

The secondary pancreatic remnant tumors that developed in the 51 patients with non-invasive IPMN at the time of initial IPMN resection were non-invasive IPMN (*n* = 26, 51.0%), invasive IPMN (*n* = 12, 23.5%) and PDAC (*n* = 9, 17.6%), whereas the secondary pancreatic remnant tumors that developed in the 37 patients with invasive IPMN at the time of the initial IPMN resection were non-invasive IPMN (*n* = 6, 16.2%), invasive IPMN (*n* = 25, 67.6%) and PDAC (*n* = 6, 16.2%) (Fig. 1). Table 2 summarizes the characteristics of the patients who underwent redo pancreatectomy after the initial IPMN resection. A median period of 37.0 months (range 1–179 months) after the initial IPMN resection, total pancreatectomy and partial resection for secondary tumors were performed in 75 patients (85.2%) and 13 patients (14.8%), respectively. No residual tumors after redo pancreatectomy (R0) were identified in 85 patients (96.6%).

**Table 2:** Redo-pancreatectomy

	*n* = 88
Redo-operation, *n* (%)	
Total remnant pancreatectomy	75 (85.2)
Partial resection	13 (14.8)
Second pathology, *n* (%)	
IPMN	69 (78.4)
Non-invasive IPMN	32 (46.4)
Invasive IPMN	37 (53.6)
PDAC	15 (17.0)
Other	4 (4.5)
Residual tumor, *n* (%)	
R0	85 (96.6)
R1/2	3 (3.4)

*IPMN* intraductal papillary mucinous neoplasm, *PDAC* pancreatic ductal carcinoma

### Timing of secondary tumor development in the remnant pancreas

The follow-up interval of surveillance for the remnant pancreas after the initial resection was every 3–4 months in 67 institutions (73.6%) and every 6 months in 19 institutions (20.9%). The total follow-up times of surveillance were over 5 years in 61 institutions (67.0%) and 5 years in 22 institutions (24.2%). All 91 institutions used computed tomography (CT) as the imaging modality for surveillance, 43 institutions (47.3%) also used magnetic resonance imaging (MRI) and/or magnetic resonance cholangiopancreatography, and 17 institutions (18.7%) used ultrasonography. Additionally, 7 (7.7%) and 3 (3.3%) institutions used endoscopic ultrasonography (EUS) and endoscopic retrograde cholangiopancreatography, respectively.

**Fig. 2:**
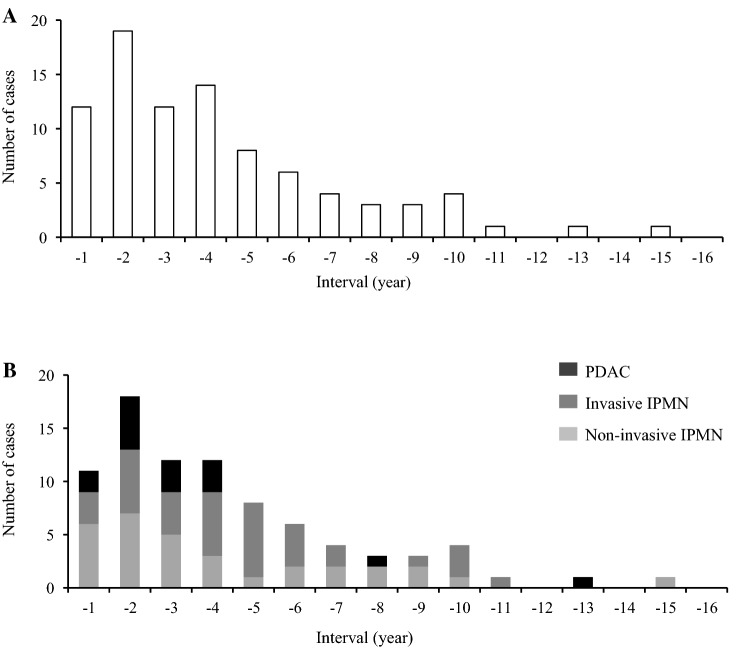
Timing of secondary tumor development in the remnant pancreas (**a**) and the breakdown of secondary tumors (**b**). *IPMN* intraductal papillary mucinous neoplasm, *PDAC* pancreatic ductal adenocarcinoma

A histogram of secondary tumor development demonstrated that most secondary tumors occurred within 1–4 years (Fig. 2a). Secondary tumors developed within 5 years and between 5 and 15 years, in 65 (73.9%) and 23 (26.1%) patients, respectively. A histogram of the number of secondary non-invasive and invasive IPMN and PDAC tumors showed no significant difference in the timing of secondary tumor development in the remnant pancreas (*p* = 0.335, Fig. 2b).

### Predictors of invasive IPMN development in the remnant pancreas

Univariate logistic regression analysis of the 11 variables revealed a significant difference only in the initial pathological diagnosis of invasive IPMN (*p* < 0.001, odds ratio 6.60, 95% CI 2.56–16.99; Table 3) and that the absence of pancreatitis had a high odds ratio with a trend toward being an independent predictor (*p* = 0.106, odds ratio 5.86, 95% CI 0.69–48.89; Table 3). Multivariate logistic regression analysis of these variables revealed that the initial pathological diagnosis of invasive IPMN was an independent predictor of secondary invasive IPMN in the remnant pancreas (*p* = 0.001, odds ratio 5.28, 95% CI 1.95–14.30; Table 3). No significant predictors for the development of PDAC in the remnant pancreas were identified (Table 3).

**Table 3:** Univariate and multivariate analyses of predictors of secondary invasive intraductal papillary mucinous neoplasm and pancreatic ductal adenocarcinoma in the remnant pancreas

Variables	Secondary invasive IPMN				Secondary PDAC	
	Univariate analysis		Multivariate analysis		Univariate analysis	
	*p* value	Odds ratio (95% CI)	*p* value	Odds ratio (95% CI)	*p* value	Odds ratio (95% CI)
Age	0.948	1.00 (0.94–1.06)			0.055	1.09 (1.00–1.18)
Gender						
Female vs male	0.130	1.98 (0.81–4.81)			0.761	0.83 (0.26–2.70)
Location of tumor						
Head vs body-tail	0.934	0.96 (0.40–2.34)			0.122	2.43 (0.79–7.53)
Residual tumor						
Absent vs present	0.888	1.14 (0.18–7.20)			0.641	102.93 ( - - - )
Cut end positive						
Absent vs present	0.906	1.12 (0.18–7.05)			0.636	110.28 ( - - - )
Pathology of the primary tumor						
Invasive IPMN vs non-invasive IPMN	< 0.001	6.60 (2.56–16.99)	0.001	5.28 (1.95–14.30)	0.828	0.88 (0.28–2.74)
Residual IPMN						
Present vs absent	0.729	1.17 (0.48–2.88)			0.204	2.40 (0.62–9.28)
Complications						
Absent vs present	0.148	2.13 (0.76–5.96)			0.506	0.66 (0.20–2.24)
Dilatation of MPD						
Absent vs present	0.897	1.06 (0.44–2.58)			0.453	1.54 (0.50–4.75)
Pancreatitis						
Absent vs present	0.106	5.86 (0.69–48.89)	0.091	6.95 (0.74–65.60)	0.128	0.30 (0.06–1.42)
Diabetes						
Absent vs present	0.666	1.21 (0.51–2.91)			0.856	1.11 (0.36–3.47)

*CI* confidence interval, *IPMN* intraductal papillary mucinous neoplasm, *MPD* main pancreatic duct, *PDAC* pancreatic ductal adenocarcinoma

## Discussion

Several studies on recurrent secondary IPMN and metachronous PDAC after IPMN resection have been reported (Table 4). While the number of target patients was higher in previous studies than in the present study, analyzing the developmental patterns of secondary tumors was challenging because of the low number of cases of redo pancreatectomy. The incidence of redo pancreatectomy for secondary tumors after initial resection for IPMN was reported as 1.4–8.5% (median 3.3%) and the actual numbers of redo pancreatectomy reported previously ranged from 3–36 (Table 4). Hence, we tried to identify the independent predictors of secondary invasive tumors by analyzing the epidemiology and pathological features of secondary tumors of the remnant pancreas after the initial resection of IPMN in 88 redo pancreatectomy cases for IPMN from a large-scale survey of institutions all over Japan.

**Table 4:** Reports of cases of recurrence in the remnant pancreas and redo-pancreatectomy

Author	Year	Initial IPMN *n*	Non-invasive; invasive (%)	Recurrence in the remnant pancreas *n* (%)		Interval period (months)	Redo-PTX *n* (%)		Invasive IPMN in redo-PTX, *n* (%)		PDAC in redo-PTX *n* (%)	
He [10]	2013	130	100: 0	22	(16.9%)	46	11	(8.5%)	3	(27%)		
Kang [11]	2014	366	81: 19	24	(6.6%)	40	5	(1.4%)	4	(80%)	1	(20%)
Yogi [20]	2015	153	77: 23	10	(6.5%)	43	6	(3.9%)	3	(50%)		
Marchgiani [23]	2015	381	78: 22	36	(9.4%)	52	9	(2.4%)	7	(78%)		
Miyasaka [24]	2016	195	82: 18	13	(6.7%)	45	10	(5.1%)	4	(40%)	4	(40%)
Hirono [18]	2016	257	67: 33	14	(5.5%)	36	8	(3.1%)	2	(25%)	2	(25%)
Blackham [19]	2017	100	100: 0	9	(9.0%)	15	3	(3.0%)				
Hirono [14]	2020	1074	77:23	70	(6.5%)	40	36	(3.4%)	16	(44%)	8	(22%)

*IPMN* intraductal papillary mucinous neoplasm, *PDAC* pancreatic ductal adenocarcinoma, *PTX* pancreatectomy

There are limited data on appropriate surveillance strategies for the remnant pancreas after IPMN resection. Moreover, the following three guidelines have different postoperative follow-up methods, potentially overlooking the early recurrence of secondary tumors. For instance, the Revision of the International Consensus Guidelines for the Management of IPMN from the IAP in 2017 recommended postoperative surveillance by CT and CA19-9 levels within 6–12 months for non-invasive IPMN. In particular, the guidelines recommended performing a cross-sectional imaging modality at least every 6 months for patients with either a family history of PDAC, a positive surgical margin with high-grade dysplasia (HGD) in the remnant pancreas, or non-intestinal pathological subtypes of resected IPMN [9]. Conversely, the 2013 European expert consensus statement recommended annual postoperative follow-up with MRI or EUS for non-invasive IPMN and compliance with the guidelines for PDAC for invasive IPMN [6]. The 2015 AGA guidelines suggested that periodic surveillance was not required for low-grade dysplasia in cystic tumors, but that follow-up with MRI should be done every 2 years for HGD or invasive cancer in cystic tumors [7]. Our study showed that approximately 70% of the institutions surveyed performed postoperative follow-up with CT and MRI every 3–4 months. This detailed and precise follow-up detected the secondary tumors in our study, and most of the secondary malignant or potentially malignant tumors were resected within 1–4 years in the institute of JSPS. Although further evidence is necessary, our data suggest that postoperative follow-up at least every 3–4 months could lead to the early detection of recurrence, which may be reflected in the prognosis of patients after IPMN resection.

In addition to short-term postoperative evaluation for the early detection of recurrence, long-term follow-up for late-onset secondary tumor development was required. Our study showed a median period of 37 months for secondary tumor detection, which was almost equivalent to 15–52 months in previous studies, as shown in Table 4. Moreover, 26.1% of the patients suffered relapse with secondary tumors, more than 5 years after initial IPMN resection. Secondary invasive IPMN tended to develop later than other tumors, after median periods of 40 months for invasive IPMN, 32 months for non-invasive IPMN, and 26 months for PDAC. Several other reports have demonstrated the importance of long-term surveillance for patients with secondary tumors more than 5 years after initial IPMN resection [5, 14, 18]. After non-invasive IPMN resection, the cumulative risk of a secondary tumor requiring surgery at 1, 5, and 10 years was 1.6%, 14%, and 18%, respectively [10]. Combined with this evidence, our data support periodic and long-term follow-up after the initial pancreatectomy. In addition to intrapancreatic recurrence during follow-up, the possibility of extrapancreatic recurrence should also be considered. Extrapancreatic recurrence from non-invasive IPMN is thought to be rare [10, 11, 14, 19–22], although metastatic recurrence of invasive IPMN to extrapancreatic organs was reported at a high rate of 45–57% [20, 23]. Thus, a protocol for long-term postoperative surveillance is required so as not to miss any recurrence after IPMN resection because the risk of intra- and extrapancreatic recurrence increases year by year.

There is limited evidence about whether secondary tumors in the remnant pancreas after IPMN resection recur like the primary tumor. The fact that secondary IPMN developed in the remnant pancreas after primary IPMN resection in 78.4% of the patients in this series suggests that secondary tumors after IPMN resection tend to be similar to the primary tumors (Table 2). Moreover, secondary non-invasive IPMNs developed in 51.0% of patients with primary non-invasive IPMNs, whereas secondary invasive IPMNs developed in 67.6% of the patients with primary invasive IPMNs (Fig. 1), suggesting that primary tumors may be a high predictor of the development of a similar secondary tumor.

Several independent predictors for a secondary tumor in the remnant pancreas after resection of initial IPMN have been reported, based on analyses of initial IPMN patients. These predictors include preoperative symptoms, tumor location (body/tail), MPD dilatation (> 10 mm) or HGD/invasive IPMN at the initial resection [14, 24]. These studies were valuable for the collection and detailed analysis of data on more patients who underwent pancreatectomy for initial IPMN. However, there were only 36 and 10 cases of redo pancreatectomy, respectively, in these studies (Table 4). Our study is unique, because it analyzed independent predictors in 88 patients who underwent redo pancreatectomy for a secondary tumor in the remnant pancreas after resection of initial IPMN, from a nationwide survey. Consistent with previous studies on independent predictors of secondary tumor development, only primary invasive IPMN at the initial resection was identified as a significant predictor (Table 3).

According to a recent study on metachronous secondary tumors after IPMN resection using targeted DNA sequencing, secondary tumors independent of the primary IPMN developed in more than half of the patients [25]. A more detailed subdivision of pathological evaluation of the primary tumors may enable us to identify a causal relationship between the primary tumor and secondary tumor types. In light of these findings, the types of secondary tumor after IPMN resection may be highly dependent on those of the primary tumor. This highlights the necessity for continued postoperative assessment of secondary tumor development in the remnant pancreas after IPMN resection.

It is noteworthy that the incidence of PDAC occurring as the secondary tumor after IPMN resection was 16–17% in the present study. A previous study similarly found the 5-year and 10-year cumulative incidences of PDAC in the remnant pancreas after IPMN resection to be 4.5% and 5.9%, respectively [24]. Surprisingly, we found that the PDAC generally developed within 5 years, although our logistic regression analysis could not specify a significant predictor of PDAC development (Table 3). However, it has been suggested that all patients who have undergone IPMN resection have high probability of PDAC development and that IPMN itself is a significant risk factor for PDAC development.

This study had some limitations. Although we collected recorded cases of patients who had undergone IPMN resection in the study period, through questionnaires from all over Japan, the number of subjects analyzed was not considerably high (88 patients). Furthermore, several clinicopathological data such as the morphologic type of IPMN, pathological grade of dysplasia, and size of tumor were not included in this study. Patients whose secondary tumor was not resected after initial IPMN resection were also not included. An increased dataset from future questionnaires will add to this model and allow for further investigation.

## Conclusion

In summary, we conducted a nationwide large-scale survey in Japan of patients who underwent redo pancreatectomy for secondary tumor development in the remnant pancreas after initial IPMN resection. The types of secondary tumors after IPMN resection correlated with those of the primary tumors, and only primary invasive IPMN was revealed to be a significant predictor of secondary invasive IPMN. Both short-interval and maximally long-term postoperative follow-up will reflect the prognosis of patients after IPMN resection. Subsequent research investigating the correlation between the primary and the secondary tumor, and the most effective follow-up method, must include prospective studies.

## Supporting information: participating institutions

Department of Surgery and Oncology, Graduate school of medical sciences, Kyushu University; Department of Surgery, Institute of Gastroenterology, Tokyo Women's Medical University Hospital, Department of Hepatobiliary-Pancreatic Surgery, National Cancer Center Hospital East; Department of Surgery, Tohoku University Graduate School of Medicine, Department of Surgery, Institute of Biomedical and Health Science, Hiroshima University; Department of General Surgery, Graduate School of Medicine, Chiba University; Department of Gastroenterological Surgery, Nagoya University Graduate School of Medicine; Division of Digestive and General Surgery, Niigata University Graduate, School of Medical and Dental Sciences; Department of Surgery, Division of Hepato-Biliary-Pancreatic Surgery, Kobe University Graduate School of Medicine; Department of Surgery, Saitama Medical University International Medical Center; Department of Surgery, Iwate Medical University School of Medicine; Department of Surgery, Kansai Medical University; Department of Surgery, Surgical Oncology and Science, Sapporo Medical University; Department of Surgery, Jichi Medical University; Department of Hepatobiliary and Pancreatic Surgery, Chiba Cancer Center; Department of Surgery, Nagasaki University Graduate School of Biomedical Sciences; Department of General Surgery and Pancreatic Surgery, Fujita Health University; Hepatobiliary-Pancreatic and Transplant Surgery, Mie University Graduate School of Medicine; Department of Surgery, Asahikawa Medical University; Department of Hepato-Biliary-Pancreatic and Breast Surgery, Ehime University Graduate School of Medicine; Department of Surgery, Oita Red Cross Hospital; Department of General and Gastroenterological Surgery, Osaka Medical College; Department of Gastroenterological Surgery, Graduate School of Medicine, Osaka University; Department of Digestive Surgery, Kawasaki Medical School; Division of Hepatobiliary and Pancreatic Surgery, Department of Surgery, Kurume University School of Medicine; Department of Surgery, Sapporo Kosei Hospital; Department of Digestive Surgery, Tenriyorodusoudanjyo Hospital; Department of Surgery, Nara Medical University; Division of Hepato-Biliary-Pancreas and Digestive Surgery in the Department of Surgery, University of Miyazaki, Faculty of Medicine; First Department of Surgery, Graduate School of Medical Science, Yamagata University; Department of Gastroenterological Surgery, Yokohama City University Graduate School of Medicine; Second Department of Surgery, Wakayama Medical University; Department of Surgery, Showa Inan General Hospital; Department of Gastroenterological Surgery, Kanazawa University Hospital; Department of Surgery, Kitasato University School of Medicine; Department of Surgery, Kyorin University School of Medicine; Department of Gastroenterological and General Surgery, St. Marianna University, School of Medicine; Department of Surgery, Chiba Rosai Hospital; Department of Hepatobiliary and Pancreatic Surgery, Graduate School of Medicine, Tokyo Medical and Dental University; Department of Hepato-Biliary-Pancreatic Surgery, Tochigi Cancer Center; Department of Surgery and Science, Graduate School of Medicine and Pharmaceutical Sciences, Toyama University; Department of Surgery, Hyogo College of Medicine; Department of Gastroenterological Surgery, Fukuyama City Hospital; Department of Surgery, Meiwa Hospital; Department of Surgery, Ashikaga Red Cross Hospital; Department of Surgery, Ibaraki Prefectural Central Hospital; Department of Gastroenterological Surgery, Iwate Prefectural Central Hospital; Department of Gastroenterological Surgery, Faculty of Medicine, Kagawa University; Department of Surgery, Teikyo University Chiba Medical Center; Department of Gastrointestinal and Pediatric Surgery, Tokyo Medical University; Department of Surgery, The Jikei University School of Medicine; Department of Surgery, Saitama National Hospital; Department of Surgery, Nakagami Hospital; Department of Gastrointestinal Surgery, Japanese Red Cross Nagoya First Hospital; Department of Gastrointestinal and Hepato-Biliary-Pancreatic Surgery, Nippon Medical School; Department of Surgery, Hachioji Digestive Disease Hospital; Department of Gastroenterological Surgery, Hirosaki University Graduate School of Medicine; Department of Gastroenterological Surgery, Hiroshima City Hiroshima Citizens Hospital; Department of Gastroenterological Surgery I, Hokkaido University Graduate School of Medicine and Department of Gastroenterological Surgery II, Hokkaido University Graduate School of Medicine
